# Advances in prime editing: Molecular innovations, Large-fragment engineering, and AI-driven design

**DOI:** 10.1016/j.bidere.2026.100104

**Published:** 2026-06-30

**Authors:** Waqar Muhammad, Xi Cao, Aziz Umar, Khan Nauman, Ziao Jiao, Junjie Liu, Xiaolong Wang, Kun Xu

**Affiliations:** a Hainan Institute of Northwest A&F University, Sanya, Hainan, 572025, China; b College of Animal Science and Technology, Northwest A&F University, No.3 Taicheng Road, Yangling, Shaanxi, 712100, China; c College of Agronomy, Northwest A&F University, No.3 Taicheng Road, Yangling, Shaanxi, 712100, China

**Keywords:** Prime editing, PE variants, pegRNA engineering, Cas9 nick, Reverse transcriptase, Large-fragment prime editing

## Abstract

Prime editing has rapidly emerged as a versatile and precise genome engineering technology capable of introducing targeted substitutions, insertions, and deletions without requiring double-strand breaks or donor DNA templates. Since its initial development, substantial progress has been achieved through iterative innovations in core components, including Cas protein variants, reverse transcriptase engineering, and prime editing guide RNA (pegRNA) design. These advancements have significantly improved editing efficiency, fidelity, and target accessibility across diverse biological systems. In parallel, the development of large-fragment editing platforms such as TwinPE, PASTE, and PrimeRoot has expanded the scope of prime editing from small sequence modifications to kilobase-scale genome rewriting. Furthermore, the integration of artificial intelligence-driven design tools has transformed pegRNA optimisation and system selection, enabling more predictable and efficient editing outcomes. This review provides a comprehensive overview of recent molecular innovations, large-fragment editing strategies, and computational approaches shaping the evolution of prime editing. Collectively, these developments position prime editing as a highly programmable and scalable platform with broad applications in medicine, agriculture, and synthetic biology.

## Graphical abstract

**Figure:**
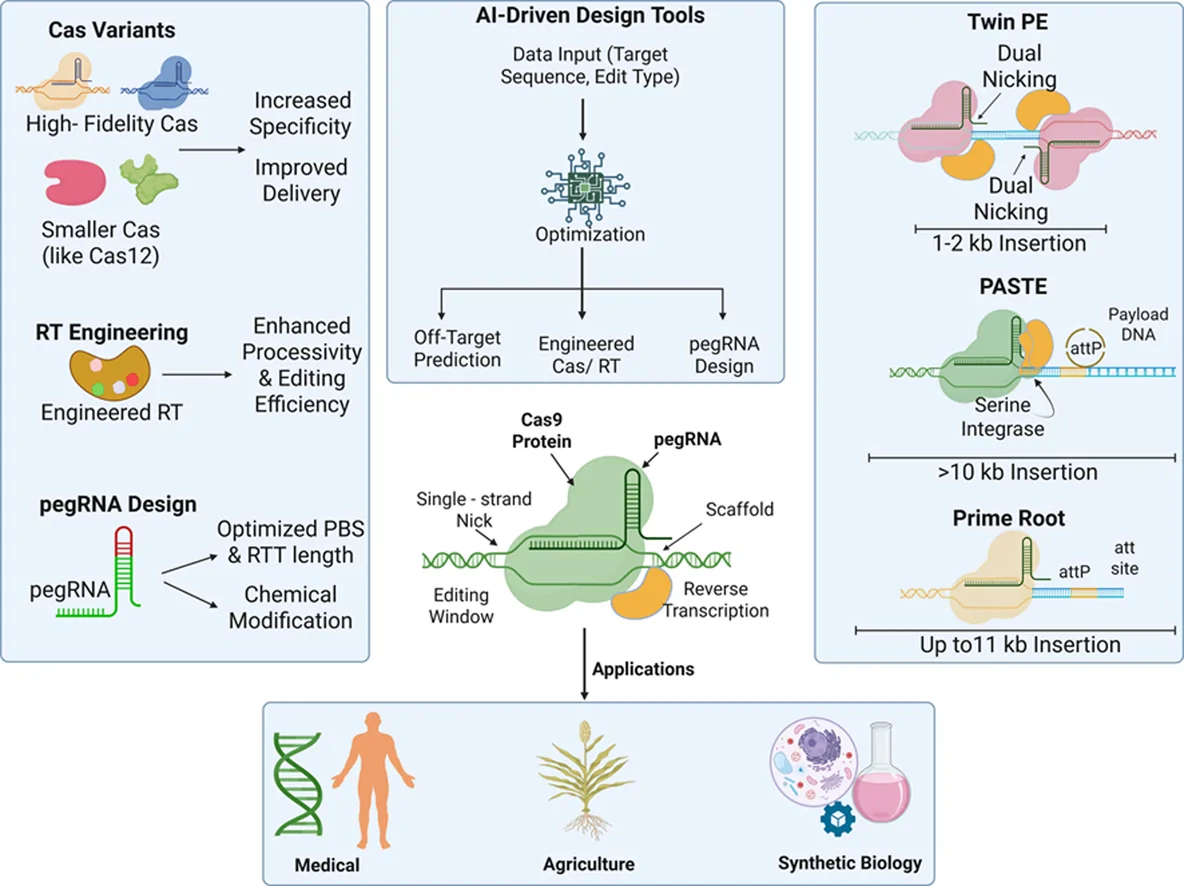

## 1 Introduction

**Fig. 1:**
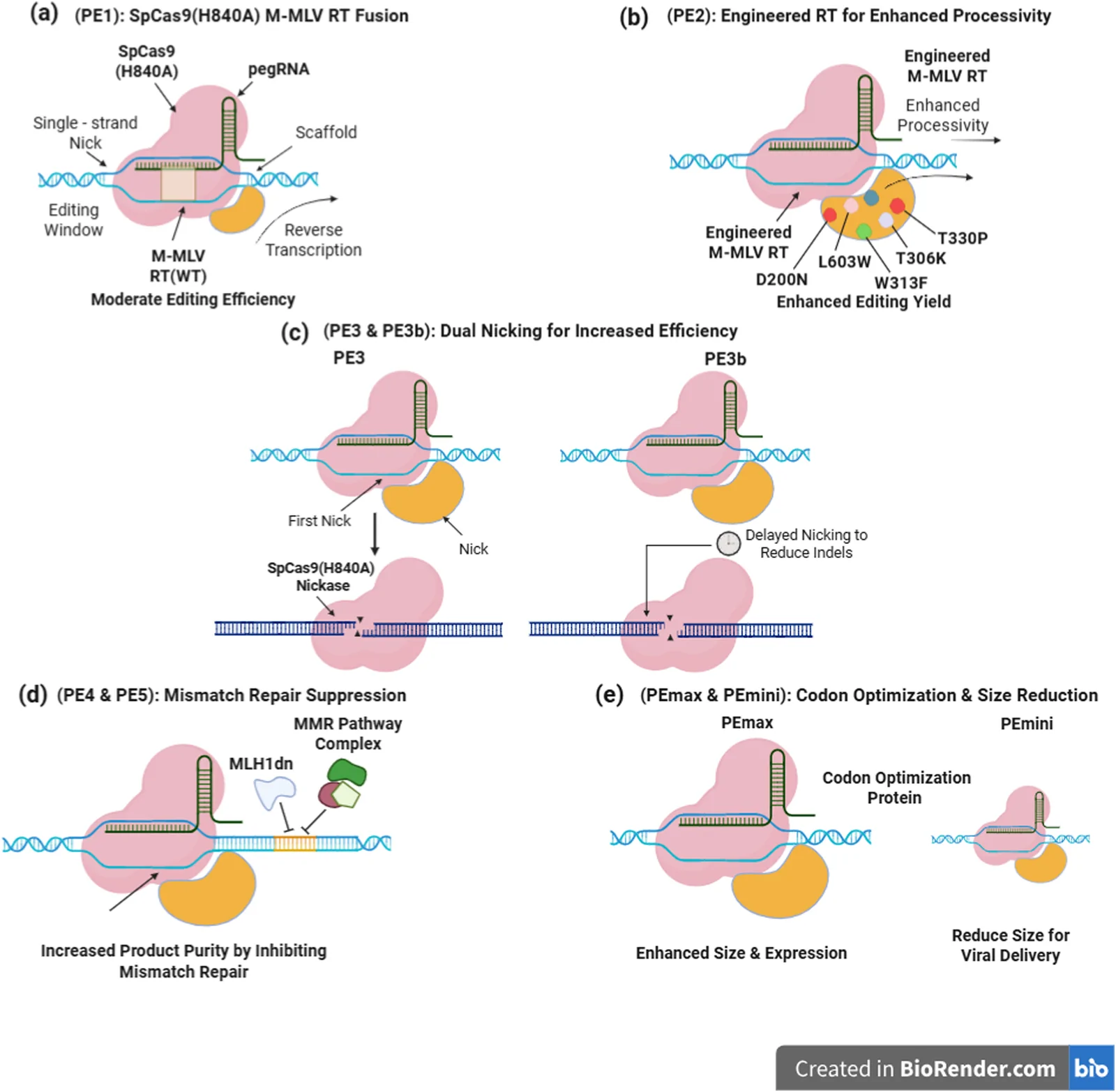
**Evolution of prime editing architectures. (a)** PE1: SpCas9(H840A) nickase fused with wild-type M-MLV reverse transcriptase. **(b)** PE2: Engineered RT improves reverse transcription and editing efficiency. **(c)** PE3/PE3b: Dual-nicking strategies increase efficiency, with PE3b reducing indels. **(d)** PE4/PE5: Mismatch repair suppression using MLH1dn improves product purity. **(e)** PEmax/PEmini: Codon-optimised and compact editors for improved expression and delivery.

Prime editing (PE) is one of the most advanced genome-editing technologies developed to date, characterised by its ability to introduce targeted substitutions, insertions, and deletions without requiring a double-strand break or a donor DNA template. The system, first introduced in 2019, has been extensively refined through the development of multiple variants that modify its core components, such as the Cas nickase, reverse transcriptase (RT), prime editing guide RNA (pegRNA), and auxiliary nicking strategies to bias the outcome of DNA repair [1]. The first version, PE1, fused wild-type Moloney murine leukaemia virus (M-MLV) RT to SpCas9(H840A) nickase, establishing the initial mechanism but showing only moderate efficiency, indicating low RT activity (Fig. 1a). PE2 improved upon this system by introducing designed mutations that boost RT processivity, thereby significantly increasing editing efficiency (Fig. 1b). In PE3, a second nick on the non-edited strand increased the editing rate but also increased indel formation. Therefore, a refined PE3b strategy was developed that reduced by-product by ensuring that the second nick occurs only after successful incorporation of the edit (Fig. 1c) [1] (see Table 1).

**Table 1:** Evolution of core prime editing architectures.

System/Variant	Key Modification	Mechanism	Editing Scope	Efficiency	Major Applications	Limitations	Ref.
**PE1**	WT M-MLV RT fused to SpCas9(H840A)	pegRNA-guided reverse transcription	Substitutions, small indels	Low-moderate	Proof-of-concept genome editing	Low RT activity	[1]
**PE2**	Engineered M-MLV RT mutations	Improved RT processivity	Small insertions, deletions, substitutions	Moderate-high	Mammalian and plant editing	Limited long insertions	[1]
**PE3**	Secondary nick on non-edited strand	Strand-biased repair	Small edits	High	Functional genomics	Indel formation	[1]
**PE3b**	Delayed secondary nicking	Conditional strand nicking	Small edits	High with fewer indels	Precise genome editing	Guide spacing constraints	[1]
**PE4/PE5**	MLH1dn mismatch repair suppression	Stabilisation of edited intermediates	High-purity substitutions	Higher vs PE2/3	Editing in MMR-proficient cells	MMR suppression concerns	[2]
**PEmax**	Codon-optimised PE2 architecture	Improved expression and nuclear localisation	Broad editing capability	High	Mammalian genome editing	Large construct size	[2]
**PEmini**	Compact Cas9-RT architecture	Size-reduced editor	Small-medium edits	Moderate-high	AAV delivery	Reduced RT processivity	[3]

As the field matured, PE4 and PE5 were developed, with PE5 incorporating MLH1dn-mediated mismatch repair suppression to improve product purity (Fig. 1d). The codon-optimised PEmax platform again advanced expression and stability, and its reduced form [2], PEmini, making it suitable for viral delivery systems that required a strict size limit (Fig. 1e) [3]. Beyond the established PE1-PE5 architectures and their subsequent optimised variants, several conceptual extensions have been proposed to further improve prime-editing performance by enhancing modulation of DNA repair pathways and integrating editing optimisation strategies. In some reports, these emerging concepts have been referred to as PE6 and PE7 [[4], [5], [6]]. However, these designations are not universally adopted within the prime-editing field and currently do not represent standardised architectures comparable to PE2, PE3, PE4, PE5, PEmax, TwinPE, or PASTE. Therefore, PE6 and PE7 are not universally adopted and should be regarded as conceptual frameworks or study-specific designations rather than established prime-editing architectures.

Besides the protein components, extensive efforts have focused on designing RNA, including the redesign of pegRNAs into stabilised variants (epegRNAs), multi-template pegRNAs, and twin-pegRNAs, enabling increased efficiency and more complex edits. Further advancements using the Cas proteins, such as modifications to longer forms of the PAM (e.g., SpCas9-NG and SpG) and smaller proteins (e.g., SaCas9 and Cas12f), expanded the repertoire of targets and delivery possibilities. These optimisations enabled prime editing to work across a broad range of organisms, including mammalian cells, plants, microbes, and model organisms. With the application of editing that extended to long-range replacements, exon replacement, and regulatory rewiring, several approaches were introduced, i.e., TwinPE, PASTE, and PrimeRoot, which became available to fill the gap between the short precision edits and the larger functional edits [[7], [8], [9], [10], [11]] (see Table 2).

**Table 2:** Engineering strategies expanding prime editing capabilities.

System/Variant	Key Modification	Mechanism	Editing Scope	Efficiency	Major Applications	Limitations	Ref.
**epegRNAs**	Stabilised RNA motifs	RTT protection	Higher editing efficiency	2-10×	Editing in primary cells	Folding context dependence	[5]
**PrimeRoot**	Plant-optimised twin pegRNAs	Dual priming	Large replacements	High	Plant genome engineering	Plant-specific constraints	[10]
**PAM-Expanded PE**	SpCas9-NG, SpG, SpRY variants	Expanded PAM recognition	Substitutions and indels	Locus-dependent	Editing AT-rich regions	Potential fidelity reduction	[26]
**Split-PE**	Split Cas9 and RT domains	Reassembly-dependent editing	Small-medium edits	High in vivo	Viral delivery strategies	Lower maximal activity	[50]
**TwinPE**	Dual pegRNAs with overlapping RTTs	Dual-flap repair	Large insertions	High	Large-fragment genome editing	Design complexity	[58]
**PASTE**	PE combined with serine integrase	Integrase-mediated insertion	Kilobase-scale insertions	High	Gene therapy	Landing pad requirement	[59]
**Multi-PBS/RTT**	Multiple priming sites	Multi-priming mechanism	Multiplex edits	High	Synthetic biology	Mis-priming risk	[74]
**High-Fidelity PE**	HF1, eSpCas9, HypaCas9 variants	Reduced off-target nicking	High-precision edits	High	Therapeutic editing	Lower activity	[132]
**Compact Cas PE**	SaCas9, Cas12f, CasX scaffolds	Small Cas architectures	Small-medium edits	Increasing	AAV or bacterial editing	RT fusion challenges	[133]

Computational design tools have co-evolved with PE technology. The design of pegRNA, the selection of a Cas variant, or the simulation of repair outcomes is now planned using AI-assisted prediction tools. Computational design tools may reduce the number of candidate pegRNAs requiring experimental evaluation; however, the magnitude of this benefit varies among studies and biological systems. Together, these discoveries have made prime editing a flexible system capable of programmable, precise editing of genomic sequences [9,12,13]. The major types of variants are reviewed according to molecular component and modifying strategy, and integrated to summarise recent developments into a single, current framework.

### 1.1 A multi-layer functional framework of prime editing systems

To provide a unified perspective on the rapidly expanding landscape of prime editing technologies, we propose a multi-layer functional framework that integrates the key determinants of editing outcomes into four interacting layers: enzyme, RNA, DNA repair, and chromatin context. The enzyme layer consists of variants of Cas and reverse transcriptase modules that provide the targeting scope, catalytic efficiency and biochemical capacity of the system. This layer determines the location and efficiency of editing, as well as the initiation and propagation of reverse transcription. The RNA layer encodes the pegRNA, which contains the PBS, reverse transcription template (RTT), and structural stabilisation elements. The function of this layer is to include information for editing and to control hybridisation dynamics, template accessibility, and overall editing precision. Editing intermediates and the decision of whether the desired edit will be kept or removed depend on the structure of the DNA repair layer. Changing the enzyme or the RNA can only have a minimal impact on editing, as processes such as mismatch repair, flap resolution, and ligation dynamics dominate the outcomes [[14], [15], [16], [17]].

The chromatin layer represents another layer of regulation that critically governs the efficiency of prime editing in cell biology, beyond the classical ones. Compared to naked DNA substrates, genomic targets are associated with chromatin structures, which either allow or block access to the editing machinery. Chromatin accessibility, nucleosome positioning, and higher-order genome organisation regulate the initial binding of Cas proteins, the formation of the R-loop required for pegRNA hybridisation, and the progression of reverse transcription. Moreover, posttranslational modifications to DNA and histones, such as DNA methylation and histone modifications (marks), may alter local DNA flexibility and trigger the recruitment of factors that regulate the efficiency of editing and the choice of DNA repair pathway. Consequently, the same editing construct can yield significantly different outcomes across different genomic regions due to differences in chromatin state [[18], [19], [20]].

Importantly, the four layers are not modular but highly interdependent. Optimisation of one layer may also cause constraints on other layers, for instance, an expanded targeting range is possible with PAM-relaxed Cas variants. Still, it could lead to a different positioning of pegRNA. At the same time, improved reverse transcriptase activity may not drive an increase in editing efficiency if editing reaction intermediates are preferentially eliminated by repair pathways. Likewise, certain favourable pegRNA designs may not work in elite closed chromatin regions, and variations in chromatin accessibility and surrounding epigenetic environment could result in different responses to modulation of DNA repair pathways. In this context, the efficiency of Prime Editing could be regarded as limited by three steps: 1) targeting limitations at the enzyme and chromatin levels, 2) limitations in synthesis at the RNA and reverse transcription level, and 3) resolution limitations at the DNA repair pathway level. This multi-layer model shows that concerted optimisation across all functional layers is needed for successful prime editing; thus, further improvements will depend on system-level engineering strategies rather than on improvements to individual functional components. This approach brings together concepts of molecular design and chromatin context/repair processes, offering a starting point for predictive, scalable genome editing [14,15,19].

### 1.2 Cas variants for PE

**Fig. 2:**
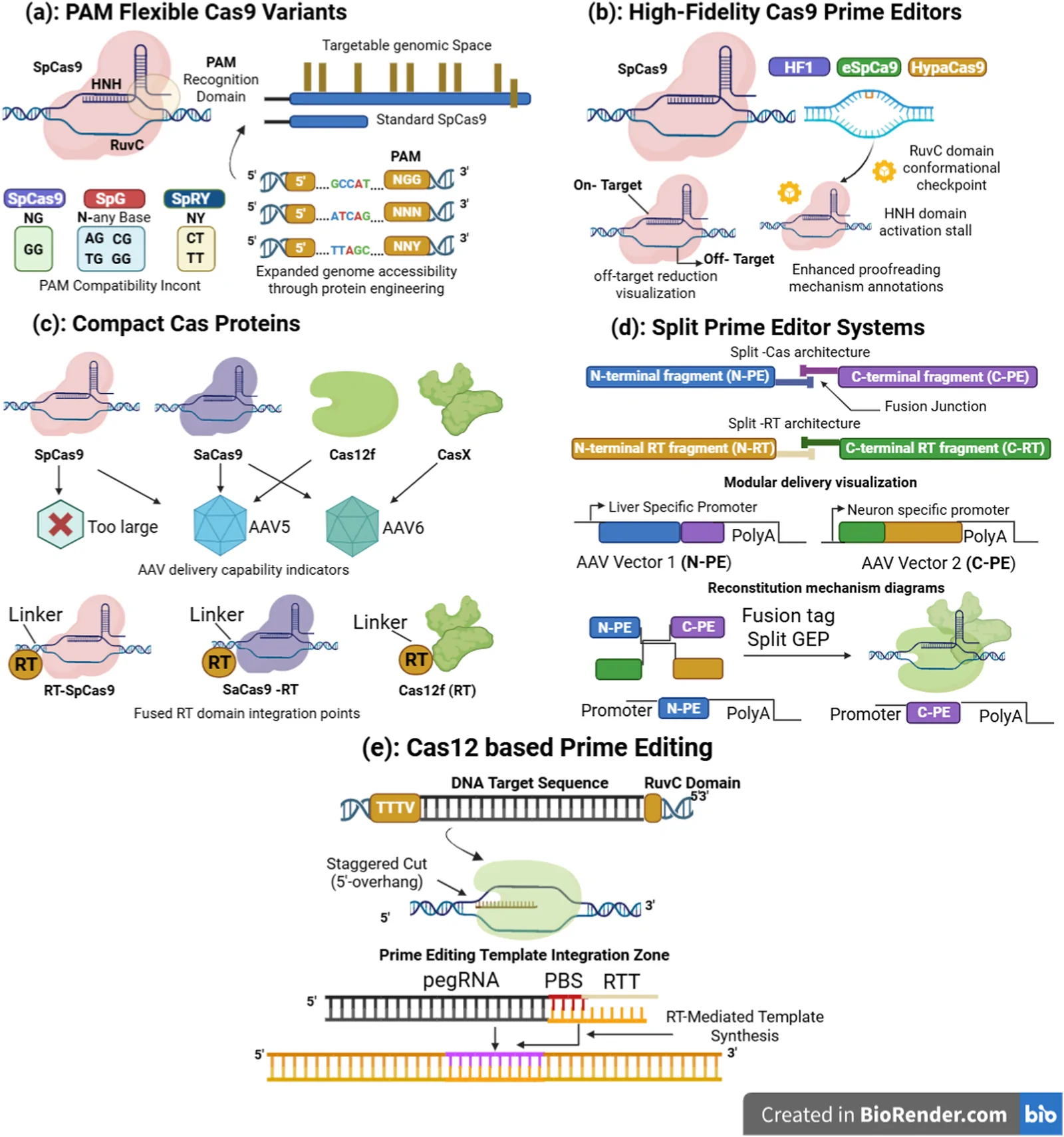
**Cas Variants Expanding Prime Editing Scope. a)** PAM-relaxed SpCas9 variants (SpCas9-NG, SpG, SpRY). **b)** High-fidelity variants (eSpCas9, HF1, HypaCas9) reduce off-targets. **c)** Compact Cas proteins (SaCas9, Cas12f, CasX) for delivery-limited systems. **d)** Split-PE systems using split-Cas9 and RT. **e)** Cas12a-based PE with staggered nicks and TTTV PAM.

Cas proteins serve as the targeting scaffold in prime editing systems, and the evolution of Cas variants has greatly broadened PE's accessibility, efficacy, and precision across diverse genomes. Initial systems used SpCas9(H840A) nickase, in which the editing location was limited by its rigid NGG protospacer-adjacent motif (PAM). To overcome this limitation, PE was adapted to several PAM-relaxed PAM-diversified variants. SpCas9-NG enabled recognition of NGN PAMs (Fig. 2a), enabling editing of pathogenic variants near non-NGG sites. Directed evolutionary methods yielded SpG and SpRY, which further relaxed PAMs, with SpRY approaching PAM-independent activity, substantially expanding the range of targetable sites. These variants proved crucial for precisely positioning the pegRNA primer-binding site (PBS) and the RTT, especially in clinically relevant genes with limited PAM availability. At the same time, PE architecture high-fidelity Cas variants were incorporated with minimal loss of efficiency. HF1, HypaCas9, and eSpCas9 derivatives that require high fidelity were effectively fused to RT domains to form PE systems with reduced off-target effects, a property highly desirable in therapeutic settings (Fig. 2b). These variants also minimise low-frequency nicking at off-target sites, hence reducing unintended repair events and insertions [[21], [22], [23], [24], [25], [26]].

Other proteins of interest have been pursued to address the delivery limitations of SpCas9; compact Cas proteins have also been explored. SaCas9(H587A) nickase has enabled packaging prime editors into mono adeno-associated virus (AAV) vectors to avoid the size limitations associated with SpCas9-based constructs (Fig. 2c). Cas12f (Cas14) and CasX (Cas12e) are also ultra-compact, but their fusion with RT domains remains to be optimised due to size considerations and catalytic anomalies. Optimisation of RT fusion domain reorientation, linker engineering, and pegRNA-binding interface optimisation for these small Cas proteins has been attempted to create PE [27,28]. PE systems have also incorporated modified Cas variants with modified DNA-binding kinetics. Engineered Cas variants with prolonged DNA residence time enhance reverse transcription efficiency by extending the editing window. Engineered variants to enhance access to chromatin, either through domain changes or including chromatin-modifying peptides, facilitate editing in densely packed genomic regions where unbinding of Cas9 is usually not possible. Another important category is split-Cas systems, such as split-Cas9 and split-RT, to deliver as one yet in a module (Fig. 2d). Splitting Cas9 provides a practical strategy for overcoming size constraints in delivery vectors, because it cleaves Cas9 into two fragments that could be assembled in cells, and would enable a tissue-specific method using a split-intein system. Split-PE systems are effective for genome editing, and the advantage is that less systemic exposure to active Cas proteins is required [4,29].

Among Cas9 derivatives, attempts to associate Cas12a with prime editing also showed promising functionality despite architectural differences in PAM recognition and cleavage. Cas12a TTTV PAM can access more AT-rich genomic regions (Fig. 2e), and its staggered cleavage enables hybrid strategies that combine with nickase engineering to rectify RT priming. Whereas Cas12a-based PEs currently exhibit limited efficiency, further engineering of pegRNA design, linker geometry, and RT fusion orientation can make Cas12a prime editors useable with AT-rich genomes [[30], [31], [32]]. Combined, the diversity of the Cas proteins has made prime editing a tool that can be tailored to the genomic context, disease treatment, and delivery constraints. Future developments are expected to focus on engineering Cas variants specifically optimised for prime editing rather than general genome editing, emphasising ease of flap formation, improved interactions with pegRNA-DNA hybrids, and the ability to tune DNA-binding dynamics to help plan efficient multi-step editing reactions. Within the multi-layer framework proposed in this review, these developments primarily enhance the enzyme layer, expanding targeting range, catalytic efficiency, and genomic accessibility [33,34].

### 1.3 Reverse Transcriptase (RT) variants

Prime editing relies on a reverse transcriptase domain that synthesises the edited DNA sequence using the pegRNA template. Several early systems used wild-type Moloney murine leukaemia virus (MuLV) RT in PE1, with the drawback of processing them with moderate efficiency for long inserts and generating truncated extension products that had to be removed from the purified product [35,36]. To enhance polymerase activity, thermostability, and template affinity, the stabilising mutations D200N, V223A, S228K, M229V, and K363N were introduced into PE2. These mutations greatly enhanced RT performance by yielding easily extendable PBS and enabling RT across diverse genomic environments. This is a reformulated version of the RT, used in future versions such as PE3, PE3b, and PEmax. Engineering efforts further yielded strains with increased strand-displacement capacity, which reduced rates of premature termination and encoded longer, more efficient RTT sequences after editing. The increase in RT binding stability to the DNA-RNA fusion and enhanced editing efficiency were observed with template modifications. Modification of the templates resulted in an increased RT binding stability to the DNA-RNA fusion and an increase in editing efficiency. This type of engineering worked particularly well with insertions >100-1000 nucleotides, which were previously inefficient at RT dissociation [[37], [38], [39], [40], [41]].

PE4 and PE5 combined to improve RT by modulating mismatch repair, using MLH1dn to block repair pathways and restore modified intermediates. In addition to dominant-negative MLH1 strategies used in PE4 and PE5 systems, alternative approaches have recently been explored to modulate the mismatch repair pathway and improve prime editing efficiency. A recent study reported the development of a small protein binder targeting MLH1, generated using computational protein design methods. This binder transiently interferes with MLH1 activity during the editing process, thereby reducing the correction of edited intermediates by the mismatch repair machinery. As a result, the incorporation of the intended edits is enhanced while maintaining controlled modulation of DNA repair pathways. This strategy represents an alternative approach to improving editing efficiency without permanently suppressing mismatch repair and highlights the potential of computational protein engineering for developing novel genome-editing regulators. Improved RT variants in these systems enable the edits to be stable enough to be resolved into the genome before the newly synthesised bases are removed by mismatch repair. This played a key role in correcting pathogenic alleles in MMR-effective cell types [[42], [43], [44]].

While some studies have explored conceptual extensions beyond PE5, including systems designated PE6 and PE7, these designations remain limited to specific experimental reports and do not constitute widely adopted prime-editing platforms. PE6 and PE7 should therefore be interpreted as emerging conceptual frameworks rather than established architectures. Proposed improvements focus on enhancing reverse transcriptase performance, improving pegRNA design, and modulating DNA repair pathways, but these have not yet been broadly validated across cell types or experimental contexts. All claims regarding PE6 and PE7 should be considered preliminary and conceptual, in contrast to well-characterised systems such as PE2, PE3, PE4, PE5, PEmax, TwinPE, or PASTE. All these next-generation editors indicate further work towards editing various biological systems more efficiently and reliably. Improved expression and nuclear localisation signals were paired with RT improvements (PEmax, codon-refined prime editor), which further improved editing efficiency. PEmini, a smaller form, removes non-essential RT sequences to reduce the size of the genetic cargo without compromising the catalytic activity, enabling the use of AAV vectors. Particular RTs have been derived that interact more effectively with pegRNA secondary structures, reducing stalling and enabling the use of long RTTs in advanced systems such as TwinPE, PASTE, and PrimeRoot [6,9,[45], [46], [47], [48], [49]].

A new category is modularised RTs, in which structural components are reconfigurable to optimise geometry using alternative Cas scaffolds. The other structural determinant of prime editing efficiency is the linker architecture (LA), which involves binding the Cas9 nickase to the reverse transcriptase domain. This linker's flexibility and length can influence the spatial positioning of the reverse transcriptase relative to the DNA substrate and pegRNA template. Optimising linker architecture may therefore improve the efficiency of reverse transcription and stabilise the editing complex, thereby enhancing editing performance. The positioning of the RT active site relative to the displaced DNA strand can be determined by optimising the custom linker during prime editing. Modularity of this kind enables integration with other Cas variants, e.g., SaCas9 or Cas12f [[50], [51], [52]]. Studies have explored RT variants fused with DNA-binding or repair-modulating peptides to promote productive flap resolution. Variants of RT that affect 5′-flap removal, 3′-flap protection, and Ligase dynamics may significantly enhance product quality and expand the range of possible edits High-fidelity RT variants: reduced tolerance to misincorporation. High-fidelity RT variants have been developed to minimise by-products, especially in therapeutic constructs that require high sequence accuracy. The next stage of RT engineering is expected to use machine-learning-directed protein design and directed evolution to optimise processivity, enhance error discrimination, and implement domain-level alterations optimised for individual Cas variants and pegRNA foldamers. These next-generation RTs will be necessary to scale up prime editing, from small-sequence editing to large-scale fragment and multi-step genome editing workflows [4,6,53].These advances further refine the enzyme layer by improving reverse transcription efficiency, processivity, and compatibility with diverse editing contexts.

### 1.4 pegRNA variant innovation

**Fig. 3:**
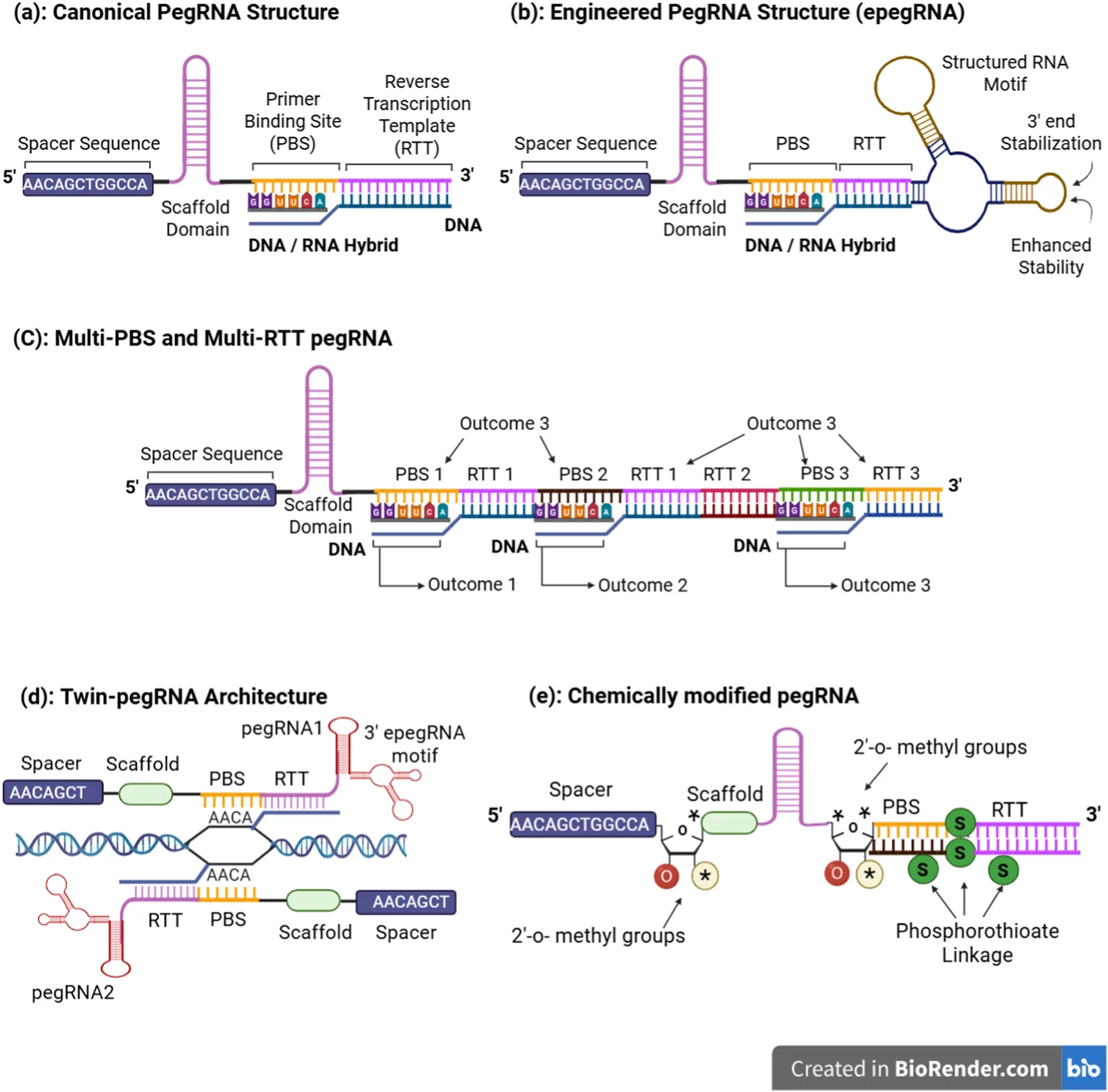
**Innovations in pegRNA and epegRNAs Design for Enhanced Editing Efficiency. a)** Conventional pegRNA (sgRNA + PBS + RTT). **b)** epegRNAs with protective xrRNA and motif. **c)** Multi-PBS/RTT pegRNAs for multi-priming or outputs. **d)** Twin-pegRNAs enabling large-fragment edits. **e)** Chemically modified pegRNAs with 2′-O-methyl and phosphonothioate groups.

Within the multi-layer framework, these innovations represent key optimisation of the RNA layer, governing template encoding, hybridisation dynamics, and editing precision. RNA components regulating prime editing, Prime editing guide RNAs (pegRNAs) are multifunctional RNAs designed to bind Cas proteins and to encode an RNA template generated by reverse transcription. Initial designs of the pegRNA incorporate PBS and RTT into the sgRNA scaffold (Fig. 3a), enabling early editing reactions in the PE1 and PE2 systems. Such unstructured extensions at 3 ends, however, were prone to degradation and poor folding, thereby limiting the efficiency in most genomic applications. These limitations prompted the design of stabilised pegRNAs, including epegRNAs, that contain stabilising RNA motifs (Fig. 3b), such as evoPreQ1 or xrRNA, to shield the RTT region. PegRNA stability has been further improved by incorporating structured RNA motifs, including xrRNA, in recent studies. Specifically, it has been demonstrated that the insertion of xrRNA motifs at the 3′ end of pegRNAs can protect the RNA against exonuclease degradation, increase intracellular half-life, and enhance prime editing efficiency. These xrRNA motif -joined pegRNA (xr -pegRNA) improves the editing yield by preserving the integrity of the RTT region, and having a more efficient reverse transcription, particularly with long template requirements or with a high turnover of RNA in the cellular environment. epegRNAs enhance editing rates in numerous cell types, especially those with longer RTTs. Additional refinement of the pegRNA was used to make the optimisation of the PBS and RTT contexts context-dependent. It was found that the length of PBS must be adjusted based on the melting temperature, GC content, and local sequence features. The RTT design needs to account for possible secondary structures and folding interactions that may interfere with RT processivity. To mitigate these limitations, systematic rules for pegRNA design were devised, resulting in improved predictability and flexibility [5,42,[53], [54], [55], [56]].

Multi-template PE builds even expanded PE activities to other priming modes with a single RNA molecule. pegRNAs with multiple binding sites, or multi-PBS (Fig. 3c), enhance priming versatility, enabling priming across regions that are either recalcitrant to hybridisation. Multi-RTT pegRNAs enable the generation of multiple edited products from a single target sequence. Recent technological advances have also enabled the design of pegRNAs by adopting versatile template RNA architectures that provide greater flexibility in encoding editing information. These have been achieved by restructuring the template regions of the pegRNA to enhance the accessibility of the reverse transcription template and by unfolding the template through an extension of the editable sequence space. This type of RNA architecture can be useful specifically in nuclease-based prime editing, where effective use of templates and precise control of repair processes are desired, thereby improving editing efficiency and expanding the range of target compatibility [57]. This allows for the simultaneous testing of mutations or the generation of complementary genotypes. A variety of specialised pegRNA derivatives enable the use of noncanonical editing strategies in which temporary sequence blockages prevent the hybridisation of the RTT until Cas binding, allowing it to be degraded or resolved through RNA secondary structure dynamics. Intentionally formed pegRNAs that are mismatched to achieve a desired result control flap activity or induce a desired outcome during repair, especially in cells proficient in MMR. To support editing on large fragment scales, RT can be extended in both directions using complementary sequences of RTT coded by twin-pegRNAs (Fig. 3d). These are twin architectures that are used to build systems such as TwinPE, PrimeRoot, and PASTE. The twin pegRNA can form overlapping DNA flaps, which can be fused to form either an insertion or replacement, making it much larger than what can be formed with single pegRNAs [46,[58], [59], [60]].

Improved pegRNA scaffolds also contain protein-binding sites that attract secondary factors to the editing site. Besides strategies to stabilise structures, other transcription systems have also been considered to enhance the functionality and expression of pegRNAs. It is important to note that engineered pegRNAs expressed by RNA polymerase II (Pol II) allow for flexible, regulated expression in cells. These systems allow the incorporation of pegRNAs into longer transcripts, which can then be cleaved into functional portions, enabling multiplex editing and providing greater control over the expression levels of the edited transcript than traditional Pol III-based systems. This technique extends the reach of prime editing, enabling precise control over transcription and the stacking of native transcription programs [61]. Other pegRNAs can bind both MCP and PP7 coat proteins, which have been fused to chromatin regulators or repair proteins to remodel the editing microenvironment. pegRNAs, as targeting small-molecule RNA-level approaches, also allow microRNA- or small-molecule-responsive pegRNAs to serve as an editing tool, enabling therapeutically controlled, cell-type-specific editing. Chemical modification of pegRNAs, such as the addition of 2′-O-methyl or phosphonothioate groups to increase pegRNA resistance to ribonuclease degradation (Fig. 3e), was developed to enhance pegRNA stability. Altered pegRNAs have enhanced editing of primary cells and in vivo, where RNA stability is typically an issue. RNA engineering exacerbates the diversity of the pegRNA designs and is essential for expanding the range of prime editing. Future technology, combining multi-template flexibility, structural stability, and context-sensitive activation in pegRNA, is designed to enable high-efficiency editing of diverse targets, including not only large-fragment insertions but also complex sequence edits [6,62,63].

### 1.5 Nicking & paired-guide variants

These strategies primarily act on the DNA repair layer, biasing repair pathways and influencing the resolution of editing intermediates. Nicking strategies and paired-guide designs are essential in defining the efficiency of editing systems, bias in strand editing, and editing repair of prime sites in prime editing systems. In the original PE2 technology, only one nick is used in the target strand, and the unedited and edited flaps of the DNA are equilibrated to incorporate the intended change. Although PE2 does not cause extensive genomic disruption, its editing efficiency is low. To improve the editing process, PE3 adds a second nick on the unedited strand, thereby biasing repair toward the edited strand. This dual nicking method is highly efficient in improving the editing process, but at a small price: the indel formation. The rationalised PE3 b system takes this strategy a step further by placing the second nick so that it comes into play only when the edited strand has been successfully synthesised, thereby minimising undesired by-products and, significantly, achieving high efficiency. Extended advances in nicking control have also been made with a nicked-RNA technology, in which altered sgRNAs or pegRNAs have been created to influence the timing and magnitude of nick formation. These methods help prevent unwanted nicking that initiates unwanted repair routes, while favouring the integration of the excised strand. Also, pegRNA masking and mismatch-priming responses regulate editing dynamics by affecting flap stability and repair pathway responses [[64], [65], [66]].

These concepts have been generalised in paired-guide techniques, which employ multiple guides to target the editing site and flexibly modify the genome with greater specificity and higher accuracy by using more than one pegRNA per locus or multiple pegRNA copies per guide. State-of-the-art systems such as TwinPE use two pegRNAs, each binding to a complementary DNA strand that encodes an RTT. This occurs in overlapping DNA sequences, where larger fragments can be combined accurately and precisely. There are several applications of paired pegRNA, such as determining which exons to keep or modify in long-range sequences, and inserting genetic cassettes into mammalian cells and plants. Taking this concept further, controlled spacing between the pegRNA and the secondary nicking guide is employed in other variants, such as PE3c, to reduce by-products, particularly in complex genomic environments. Other paired-nicking methods rely on programmable timing devices to enable sequential, rather than simultaneous, modification of the strands, such as variations in promoter strength or RNA stability. Also, apart from the nick-and-repair systems, nucleases, and prime editors, more accurate and efficient systems have emerged recently. These nuclease prime editors are even better, as they feature: These systems provide greater flexibility in prime editing and offer an alternative strategy to increase prime editing efficiency with tolerable precision by tuning cleavage activity and activating repair processes [61,[67], [68], [69]].

Hybrid systems like PASTE combine pegRNA-mediated priming with serine integrases or recombinases, which enable long sequences of DNA to be inserted in a hybrid fashion; the combination of target copying and site-specific recombination. In this method, pegRNAs are used to install landing sites (attB/attP), and a large DNA cargo is inserted in an integrated form with the assistance of integrase, without forming a double-strand break. Likewise, paired pegRNAs are used in PrimeRoot to promote the replacement of a large DNA fragment in plant systems infected by conventional, inefficient homologous recombination methods. Moreover, even fewer collateral activity variants of nickase are used to prevent unintended cleavage further. To compromise on editing efficiency and minimise indel formation, dual-nickase systems have been proposed in which the nick positions are offset. Together, nicking and paired-guide methods can expand the range of prime editing by allowing greater control over strand editing, repair pathway bias, and editing outcomes. These methods are also anticipated to be further optimised to enhance the efficiency and fidelity of genome engineering in several biological systems [[70], [71], [72], [73]].

### 1.6 AI prediction & computational PE design

The use of artificial intelligence (AI) has optimised prime editing by predicting pegRNA efficiency, sequence compatibility, repair dynamics, and optimal variant combinations. Manual selection of PBS and RTT before design was based on empirical heuristics. With the availability of large amounts of editing outcomes, machine learning methods have begun to learn more intricate relationships between sequence functions. Current AI technologies consider thousands of predictive features, such as GC content, target-site secondary structure, chromatin accessibility, nucleosome occupancy, and the melting temperature of the hybrid between PBS and DNA. DeepPE is a deep learning prediction model, whereas PrimeDesign and PE-Designer are computational design platforms that assist in pegRNA generation and experimental planning. These tools differ substantially in methodology and should not all be classified as artificial intelligence systems. These tools are used to estimate the optimal performance of PBS lengths and RTT structure types in reducing by-products [74,75].

Generative models extend this by optimising pegRNA design using stochastic methods. Rather than ranking the existing designs, generative algorithms generate entirely novel pegRNAs that optimise multiple parameters simultaneously, including efficiency, stability, minimality of secondary structure, and compatibility with the Cas-RT fusion. These tools will reduce experimental design time by lowering the quantity of pegRNA variants required to validate experimentally. AI is also used in selecting among Cas variants. The most useful PAM-relaxed editors are SpG and SpRY, which are useful when the desired edits lack convenient PAMs. Computational approaches may assist in selecting candidate Cas variants for experimental evaluation, particularly in cases where high-fidelity Cas proteins are most useful for specificity. Optimisation of split-PE systems and small Cas nucleases for AAV delivery is also underway, and computational approaches may help evaluate sequence constraints and predicted cutting dynamics for each scaffold [[76], [77], [78]]. Beyond editor components, AI-guided protein engineering has also been applied to the recombinases used for large-fragment integration: high-throughput profiling combined with AI-engineered Cre variants (the PCE/RePCE systems) markedly improved recombination efficiency and enabled scar-free, programmable chromosome engineering across kilobase-to-megabase scales in both plant and human cells [79].

**Fig. 4:**
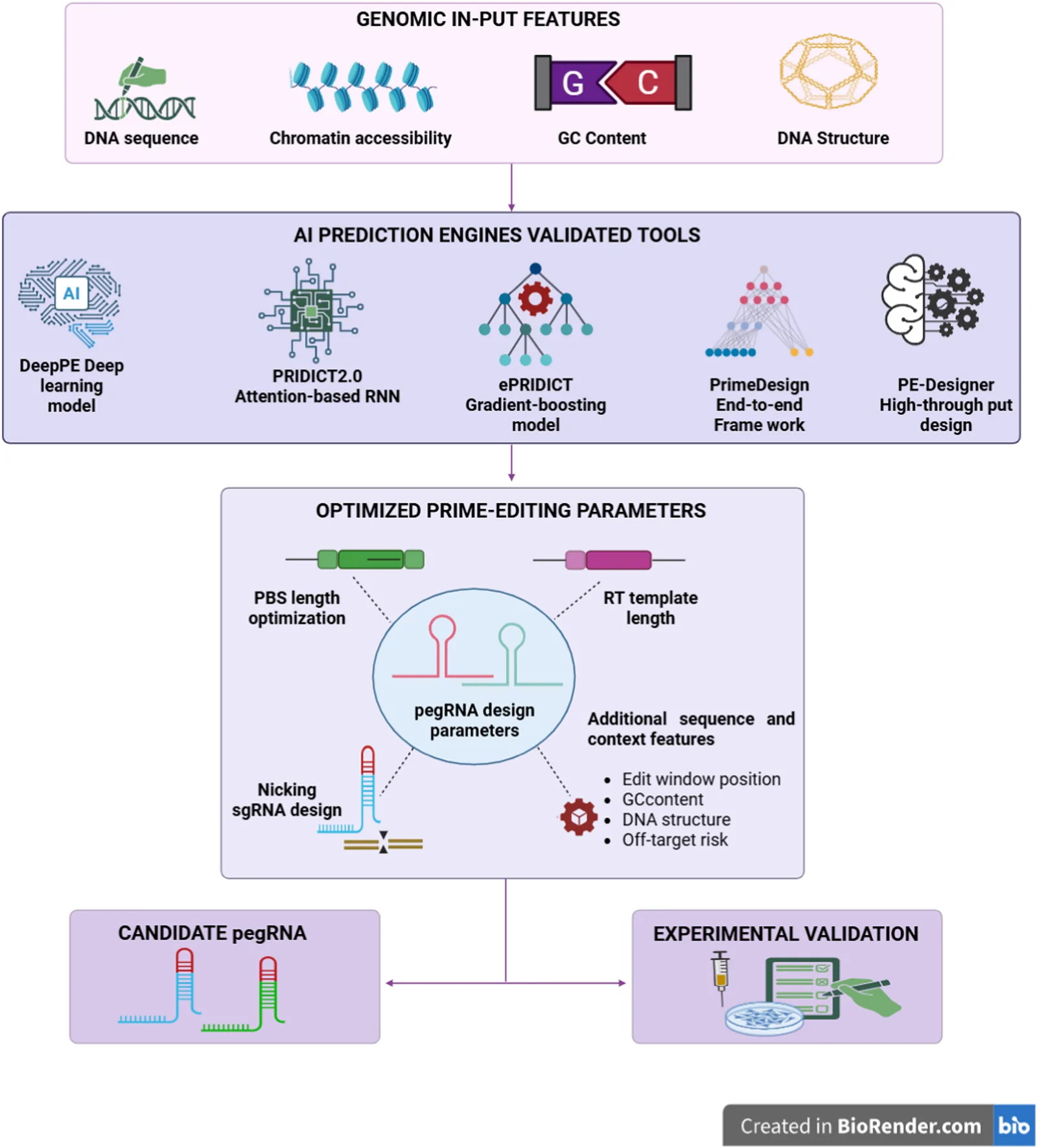
**Computational and AI-assisted approaches for prime-editing design.** Experimentally validated computational tools include deep-learning models for editing-efficiency prediction and rule-based pegRNA design platforms. These approaches assist candidate prioritisation, pegRNA design, and editing-outcome prediction. Dashed arrows indicate emerging or conceptual applications, including chromatin-aware prediction models and future automated optimisation workflows, which currently require further experimental validation.

Computational modelling is also useful in selecting reverse transcriptase. Computational models have been explored to estimate RT-pegRNA compatibility, the likelihood of long-template completion, and the likelihood of synthesis stalling due to secondary structure. This informs the application of Engineered reverse transcriptase variants in editing activities that involve large fragments. Paired-guide and large-fragment PE strategies are especially difficult to design manually; thus, Computational design tools provide guidance and prioritisation but still require experimental validation. TwinPE design software computes the optimal distance between pegRNA, the length of the RTT overlap, and the priming directionality. PE structures such as PASTE, which consist of large-scale fragments, require precise placement of landing pads, integrase recognition sequences, and PE-encoded junctions. Computational tools may assist the design of complex multi-component systems by prioritising candidate configurations for experimental testing. Another application of machine learning to PE is predicting repair pathways. Computational studies have explored repair-outcome prediction; however, accurately predicting repair pathway utilisation remains challenging. Based on these predictions, PE4/PE5 variants with MLH1dn are used, or a simple PE3b configuration is chosen. The further advancement of AI is expected to introduce real-time sequencing feedback obtained in automated laboratories, where closed-loop optimisation systems enable continuous model training on novel editing datasets. Future computational frameworks may incorporate automated optimisation strategies; however, reinforcement learning and closed-loop systems remain largely conceptual and have not yet been widely experimentally validated. This feedback of computational prediction and experimental validation will make prime editing design an automated procedure to a significant extent. The AI-assisted computational workflow is summarised in Fig. 4, where genomic input features, including target sequence, chromatin accessibility, and secondary structure, are processed through machine-learning prediction engines to yield optimised prime editing parameters such as RT variants, PBS length, and nicking strategy [[80], [81], [82], [83], [84]].

The latest research has focused on developing standardised toolkits to facilitate more thorough assessment and optimisation of prime editing systems. An example is the TRU-PE (Trackable Universal Prime Editor) platform, a modular system for tracking the results of prime editing across multiple genomic loci. This system implements reporter-based modules that enable it to track editing events easily and to quantitatively compare pegRNA designs and editing configurations. TRU-PE enables scalable genome engineering by simultaneously evaluating the efficiency of editing a single or multiple target site and improving the reproducibility of experimental results. These toolkits are especially useful for optimising high-throughput pegRNA architecture, variants of editors, and target sites, and for scaling up the generation of next-generation prime editing applications [85]. In addition to optimisation, Computational approaches are increasingly being incorporated into prime-editing workflows; however, not all available tools can be classified as artificial intelligence (AI). Current computational resources can be broadly divided into four categories: deep-learning prediction models, machine-learning outcome prediction models, rule-based pegRNA design tools, and integrated computational design platforms. Deep-learning models such as DeepPE, PRIDICT2.0, and ePRIDICT are trained on large experimental datasets and are primarily used to predict prime-editing efficiency, product purity, or editing outcomes from sequence-derived features. These models employ neural-network architectures capable of identifying complex relationships among pegRNA architecture, genomic sequence context, and editing performance [34,40,76,86].

In contrast, rule-based design tools such as PrimeDesign do not rely on machine learning. Instead, they apply predefined biological constraints and design rules to generate pegRNAs and nicking guide RNAs (ngRNAs) for user-specified edits. Similarly, computational platforms such as PE-Designer facilitate guide design, sequence analysis, and experimental planning but should not be interpreted as predictive AI systems. Distinguishing predictive models from design platforms is important because their underlying methodologies, capabilities, and limitations differ substantially. While predictive models attempt to estimate editing outcomes, design platforms primarily assist in guide generation and experimental workflow optimisation. To facilitate comparison among currently available computational tools for prime-editing design, representative platforms are summarised in Table 3. These tools differ substantially in their methodological framework, predictive objectives, and practical applications, ranging from pegRNA design and candidate prioritisation to editing-efficiency and editing-outcome prediction [66,87].

**Table 3:** Representative computational tools for prime-editing design and prediction.

Tool	Category	Model Type	Primary Function	Input	Output	Validation Status	Key Limitation	Reference
**PRIDICT2.0**	Deep-learning prediction model	Deep neural network	Predict editing outcomes and efficiency	Sequence and pegRNA features	Predicted editing outcome and efficiency	Independent validation reported	Primarily trained on mammalian datasets	[38]
**ePRIDICT**	Deep-learning prediction model	Deep neural network	Predict editing efficiency and product purity	Sequence and pegRNA features	Predicted editing outcome metrics	Independent test datasets reported	Limited data available for large-fragment editing	[38]
**PrimeDesign**	Rule-based design platform	Rule-based algorithm	Generate pegRNA and ngRNA designs	Desired edit and target sequence	Candidate pegRNA and ngRNA designs	Widely used design platform	Does not directly predict editing efficiency	[66]
**Easy-Prime**	Machine-learning-assisted design tool	Machine-learning-assisted ranking	Candidate prioritisation	Sequence and editing features	Ranked candidate pegRNAs	Validation reported by original study	Performance may vary among biological contexts	[75]
**PE-Designer**	Computational design platform	Rule-based computational workflow	PBS/RTT design and candidate ranking	Target sequence and desired edit	Candidate pegRNA designs	Experimental design support	Limited predictive capability	[87]
**DeepPE**	Deep-learning prediction model	Convolutional neural network (CNN)	Predict prime-editing efficiency	Target sequence, pegRNA features	Predicted editing efficiency	Experimentally evaluated by original study	Limited validation across diverse cell types and organisms	[88]

### 1.7 Generalizability and limitations of AI-assisted prime editing design

Despite recent advances, several limitations restrict the broad application of AI-assisted prime-editing design. Most currently available prediction models have been trained using datasets generated in a limited number of mammalian cell lines, particularly HEK293T, K562, and HCT116 cells. Consequently, model performance may decline when applied to primary cells, embryos, livestock species, plants, microorganisms, or in vivo systems. Furthermore, available datasets are heavily enriched for substitutions and small insertions or deletions, whereas relatively few datasets exist for large-fragment prime-editing systems such as TwinPE, PrimeRoot, or PASTE. Therefore, models trained on small-edit datasets may not accurately predict outcomes associated with complex editing architectures [40].

Other factors that will affect the efficiency of editing will involve chromatin accessibility, methylation status of the genome, local sequence composition, genome repair pathway activity, levels of editing protein and delivery. These factors can differ in organisms and cell types and predict performance may not be the same for all cells and organisms. Furthermore, most of the deep-learning models are used mostly as "black-box" predictors, failing to offer much mechanistic information about the biological processes that lead to editing outcomes. Hence, the predictions using computation should be treated as complementary tools used to help prioritise candidates for further study, not as experimental alternatives. The available computational tools have a range of predictive capability, methods and reported performance as summarised in Table 3. The main difference between the two is that the deep-learning based platforms are used for editing efficiency or outcome prediction, while the rule-based based platforms focus on pegRNA generation and experimental planning [2,89].

Another important limitation is dataset imbalance. Most currently available prime-editing datasets are enriched for substitutions and small insertions or deletions, whereas relatively few examples exist for large-fragment prime-editing systems such as TwinPE, PrimeRoot, or PASTE. Consequently, models trained primarily on small-edit datasets may not accurately predict outcomes for complex architectures or paired-pegRNA designs. Biological factors such as chromatin accessibility, DNA methylation, local sequence composition, DNA repair pathway activity, editor expression levels, and delivery method can strongly influence editing efficiency. Since these vary among cell types, organisms, and experimental conditions, predictive performance may not generalize. Most deep-learning models function as “black boxes,” providing limited mechanistic insight into why a particular pegRNA or configuration is predicted to succeed. Developing interpretable models that can reveal mechanistic rules underlying editing outcomes remains an important future direction. Therefore, computational predictions should be regarded as complementary tools for candidate prioritisation, not substitutes for experimental validation. Users must exercise caution when applying models to new loci, novel cell types, or uncommon edits. In practice, computational predictions are most effective when integrated with iterative experimental validation. A typical workflow involves computational prioritisation of candidate pegRNAs, experimental testing of the highest-ranked designs, and incorporation of experimental outcomes into subsequent model refinement. Such closed-loop approaches may improve predictive performance over time; however, fully autonomous optimisation systems have not yet been broadly demonstrated for prime-editing applications. Consequently, comparisons among tools should be interpreted cautiously because training datasets, validation strategies, and biological contexts differ substantially among studies [42,76,90,91].

### 1.8 Large-fragment prime editing

Large-fragment prime editing aims to extend the capabilities of prime editing systems to enable the insertion or replacement of DNA sequences ranging from hundreds to thousands of base pairs. Early implementations of prime editing were largely limited to small insertions (typically <50 bp) due to constraints on RT processivity, pegRNA instability, and difficulties resolving long DNA flaps during repair. To overcome these limitations, several advanced systems have been developed that specifically enable large-scale genome modifications. Among these, TwinPE, PASTE, and PrimeRoot represent key platforms that address distinct bottlenecks in large-fragment editing [10,58,59].

**Fig. 5:**
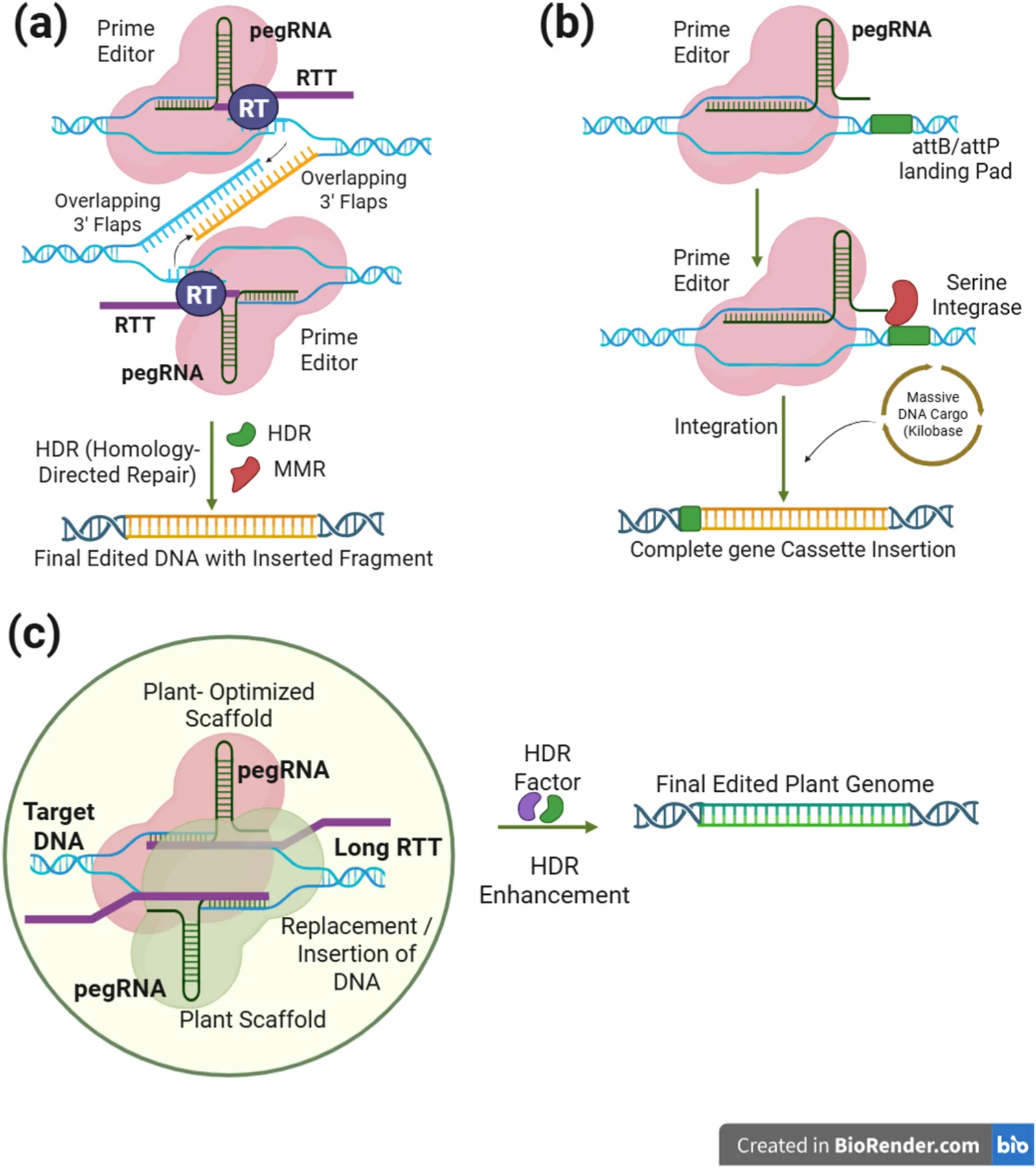
**Overview of large-fragment prime-editing strategies.** (a) TwinPE uses paired pegRNAs targeting opposite DNA strands to generate overlapping 3′ DNA flaps that facilitate precise insertion or replacement of larger DNA fragments. (b) PASTE combines prime editing with serine integrase-mediated DNA insertion, allowing efficient integration of kilobase-scale cargo after installation of an integrase recognition site. (c) PrimeRoot adapts paired-pegRNA prime editing for plant genome engineering through plant-optimised editing components and repair strategies that facilitate targeted replacement of larger genomic regions.

TwinPE utilises two pegRNAs targeting opposite DNA strands, each encoding a complementary RTT. This results in the generation of overlapping DNA segments that form long 3′ flaps, which are subsequently resolved during repair. This architecture significantly increases the maximum insertion size and enables high-precision exon replacement and cassette insertion without introducing double-strand breaks (Fig. 5a). The overlapping 3′ DNA flaps generated by the two pegRNAs provide complementary repair intermediates that can be resolved into a continuous edited sequence. This overlap stabilises the repair process and reduces dependence on a single long reverse-transcription event, thereby facilitating precise insertion or replacement of larger DNA fragments. TwinPE has demonstrated the ability to install gene fragments exceeding 100 bp in mammalian cells efficiently and has also been used to rewire regulatory elements [58]. PASTE further expands large-fragment editing capabilities by combining prime editing with serine integrases. In this system, a pegRNA first installs a genomic landing pad containing attB or attP sites, after which an integrase mediates the insertion of large DNA cargo, including sequences of several kilobases (Fig. 5b). This hybrid strategy bypasses the limitations of RT-mediated synthesis of long sequences. Rather than requiring reverse transcriptase to synthesise the entire DNA cargo, PASTE uses prime editing only to install the integrase recognition sequence. The subsequent serine integrase-mediated insertion step enables efficient integration of kilobase-scale DNA fragments while avoiding many of the processivity constraints associated with long reverse-transcription templates. PASTE has been successfully applied to integrate therapeutic-scale gene cassettes into mammalian cells, demonstrating high specificity and low off-target integration [59].

A more recent advance is the emergence of large-fragment platforms that achieve multi-kilobase insertion entirely without double-strand breaks or recombinases, removing the landing-pad requirement of PASTE. QuadPE coordinates four engineered pegRNAs two nicking the genomic locus and two nicking the donor who's complementary 3′ flaps anneal to drive directional integration of up to 26 kb, with the gPAM-out configuration outperforming gPAM-in by ∼1.7-fold and achieving ∼51% insertion at the TRAC locus in primary human T cells. Prime Assembly (PA) uses twinPE-generated ∼35-nt genomic flaps to capture PCR-tailored linear dsDNA donors and can even assemble overlapping fragments in a Gibson-like in-cellulo reaction enabling insertion of cargos up to 11.3 kb, including the full-length DMD cDNA at AAVS1. PRIME-In generates a primed-microhomology flap on the donor to template endogenous polymerase extension, reaching 9.6 kb; an evolved PRIME-In 2.0 with a complementary-strand nick gives a further 1.4-2.4-fold gain and integrated a 3-kb CD19 CAR cassette at ∼47% efficiency in primary T cells. Notably, QuadPE and PA remain active in cell-cycle-arrested and non-dividing cells, underscoring the translational advantage of a DSB- and HDR-independent mechanism [[92], [93], [94]]. PrimeRoot extends large-fragment prime editing to plant systems, where homologous recombination-based approaches are inefficient. This platform employs paired pegRNAs with optimised plant-specific scaffolds and long RTTs to guide targeted insertion or replacement of extended DNA fragments (Fig. 5c). It has enabled functional gene repair in crop species without introducing transgenes, thereby advancing plant genome engineering applications. Plant-specific optimisation of pegRNA architecture, promoter systems, and repair pathway engagement contributes to improved editing outcomes. These adaptations help overcome the generally low efficiency of homologous recombination in plants and facilitate targeted replacement of larger genomic regions [10].

Besides these innovations, platform-specific molecular components have also been improved, enhancing large-fragment editing. Single variants of RT engineered with greater processivity permit longer RTT templates to be synthesised. Programmed pegRNAs that have built-in structure are used to stabilise long templates and lower the number of suppressive secondary structures. PegRNA and multi-RTT designs can encode segmented templates, reducing RT stalling during long-range DNA synthesis. There is also optimisation of cellular repair pathways that improve the efficiency of large-fragment integration. MMR inhibition, formation of HDR-like processes, and regulation of flap endonuclease activity are strategies that favour stabilising and ligating long DNA flaps. Although chromatin remodelling solutions, such as Cas-RT fusion or pegRNA aptamer recruitment, enhance cross-linking of densely packed genomic regions [68,[95], [96], [97]].

Computational approaches have been explored to assist the design of large-fragment prime-editing systems by evaluating pegRNA spacing, RTT length, and candidate integration sites. However, predictive modelling for large-fragment editing remains less mature than models developed for small-edit prime-editing applications, and experimental optimisation continues to play a central role in system development. They are predictions of the optimal spacing of pegRNA, the RTT length, the flap-overlapping structure, and the choice of integration sites. Flap dynamics and repair outcome modelling enable the design of editing components in a coordinated manner to efficiently perform multi-step genome rewriting. And as the future of RT engineering, pegRNA design, paired-guide coordination, and computational optimisation continue to improve, it is becoming feasible to use large-fragment prime editing with relative ease to engineer complex genomes. These advances enable applications such as exon replacement in genetic diseases, insertion of custom regulatory elements, and insertion of therapeutic gene cassettes, and they enable a range of specific and deep-level genome rewriting [76,87,98]. Large-fragment editing strategies require coordinated optimisation across multiple layers, particularly the enzyme, RNA, and DNA repair layers, highlighting the importance of system-level design.

### 1.9 Prime editing in the context of other genome-editing technologies

Prime editing complements other genome-editing technologies, including base editing and CRISPR-Cas9-mediated homology-directed repair (HDR). Base editors generally exhibit high efficiency for transition mutations but are limited to specific nucleotide conversions and cannot readily introduce targeted insertions or deletions. HDR-based genome editing enables large DNA insertions but requires double-strand DNA breaks and donor DNA templates, which can increase undesired repair outcomes and reduce efficiency in non-dividing cells. In contrast, prime editing enables targeted substitutions, insertions, and deletions without requiring double-strand breaks or donor templates. However, prime editing often exhibits lower editing efficiencies than base editing and remains technically challenging for very large DNA insertions. Therefore, these technologies should be regarded as complementary approaches whose suitability depends on the desired editing outcome and biological context 1,42.

### 1.10 Applications of modern prime editing variants

**Fig. 6:**
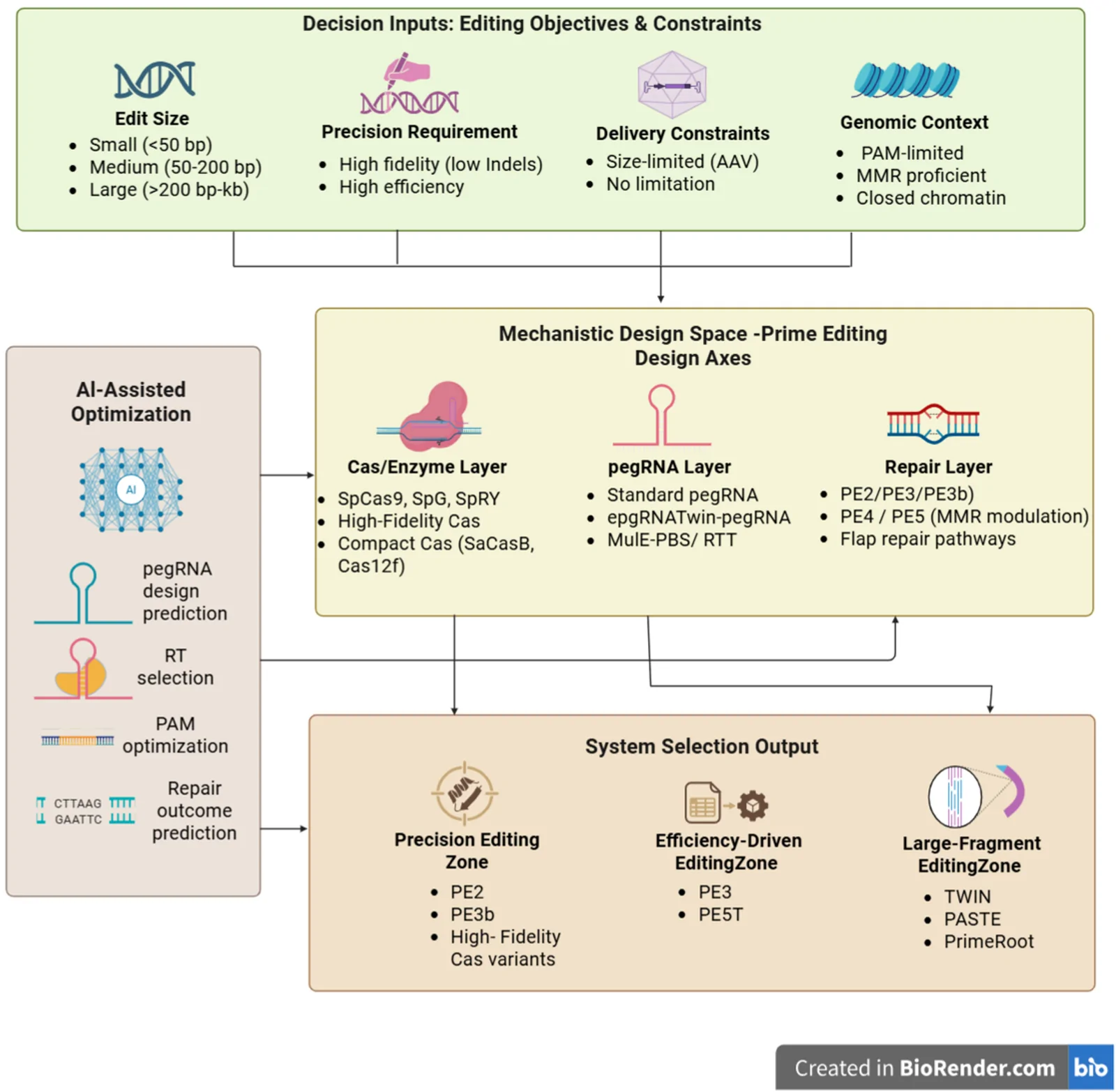
**Decision framework for selecting prime editing strategies based on editing goals and biological constraints.** Small edits (<50 bp) with high precision requirements favour PE2 or PE3b systems, while efficiency-critical applications may benefit from PE3 or PE5. Large-fragment insertions require TwinPE or PASTE, depending on whether integrase-mediated insertion is acceptable. Delivery constraints guide the selection of compact or split prime editors. Computational design tools may assist pegRNA selection and candidate prioritisation, although experimental validation remains necessary for optimal system configuration. Computational design tools shown in the workflow represent decision-support systems rather than fully autonomous optimisation platforms.

**Fig. 7:**
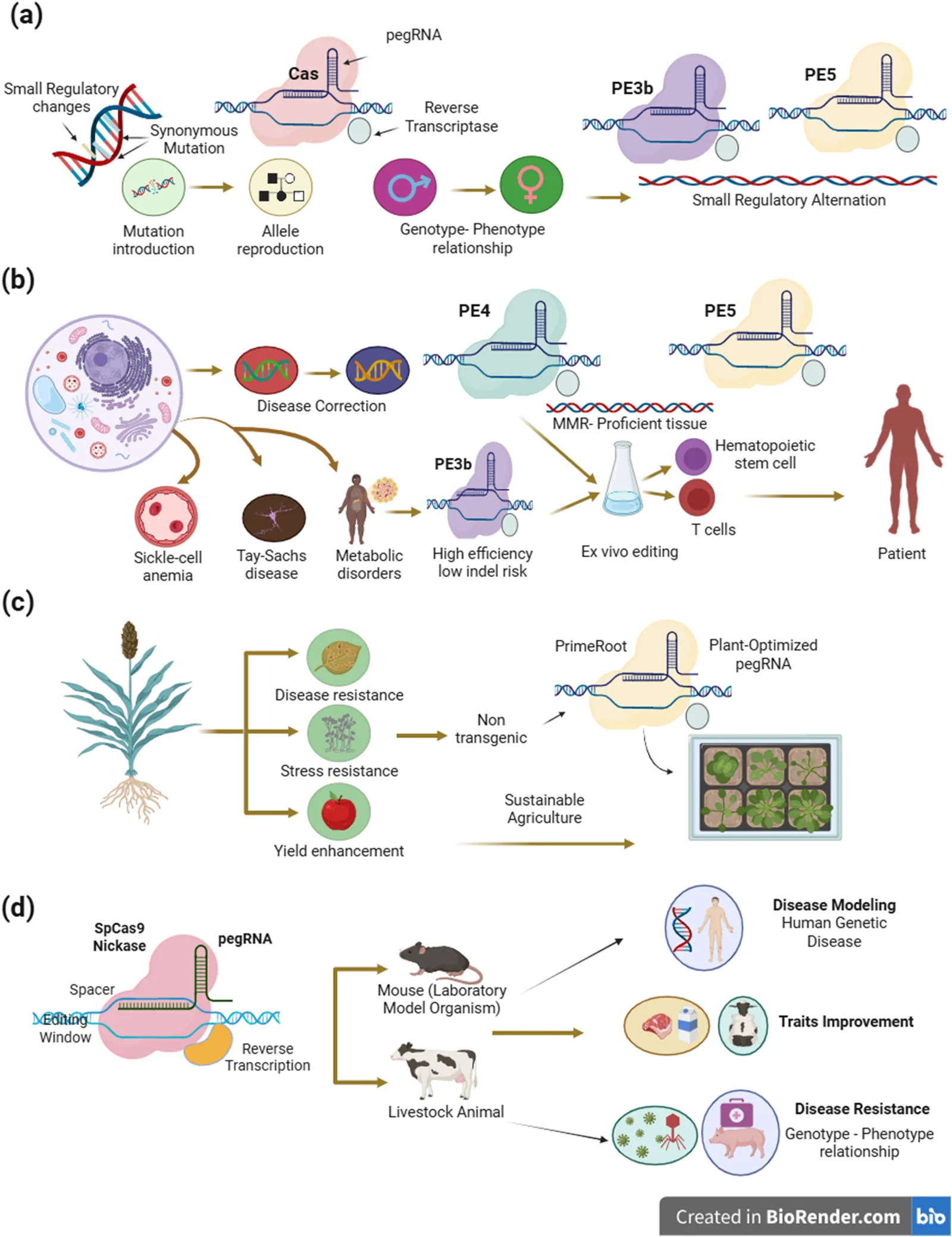
**Applications of prime editing in different biological systems. (a)** Precise introduction of small regulatory mutations for studying genotype-phenotype relationships. **(b)** Therapeutic applications including correction of disease-causing mutations and ex vivo editing of hematopoietic stem cells and T cells. **(c)** Plant genome engineering for crop improvement, including disease resistance, stress tolerance, and yield enhancement using PrimeRoot and plant-optimised pegRNAs. **(d)** Animal genome editing for disease modelling.

To facilitate practical implementation, prime editing system selection can be guided by key parameters including edit size, required precision, delivery constraints, and cellular repair context (Fig. 6). Small, high-fidelity edits are best achieved using PE2 or PE3b systems, whereas efficiency-driven applications may benefit from PE3 or PE5. Large-fragment modifications require specialised platforms such as TwinPE or PASTE. This framework highlights how context-dependent selection of prime editing strategies enables an optimal balance between precision, efficiency, and scalability. The growing family of advanced prime editing systems has simplified its use across basic research, biotechnology, agriculture, and medicine. PE has been used in functional genomics to introduce mutations accurately enough (Fig. 7a) to reproduce alleles of human diseases and examine the relationship between genotype and phenotype in some detail. Better pegRNA design and PAM-expanded forms of Cas can be used to edit at loci previously inaccessible, enable saturation mutagenesis, dissect regulatory motifs, and perform combinatorial perturbation experiments. PE3b and PE5 systems, which form minimal indels, are critically useful for the realisation of small regulatory alterations or synonymous changes that demand high product purity [72,[99], [100], [101]].

PE can be used to introduce subtle changes to a genomic architecture with synthetic biology applications without distorting it using DSBs. Small regulatory elements, a PE2 Special promoter-binding sequence or a protein label can be ligated to PE2, PEmax, or Engineered prime editing guide RNAs (epegRNAs) stabilised systems without destabilising the scaffolds. Large-fragment prime-editing technologies, such as TwinPE and PASTE, can insert synthetic circuits, multi-gene assemblies, or engineered receptors into their native genomic context. In therapy, prime editing has considerable promise in preclinical studies for correcting pathogenic variants in monogenic diseases (Fig. 7b) [[102], [103], [104]]. PE4 and PE5 that inhibit mismatch repair to stabilise edited intermediates and promote uniformity in the products of MMR-proficient tissue. The variants of high-fidelity Cas dramatically reduce the risk of off-target effects, and PEmini enables compatibility with viral vectors. Preclinical models have been used to test optimised PE systems for the treatment of diseases such as sickle cell anaemia, Tay-Sachs disease, and metabolic diseases. PE3b systems offer a favourable balance between high efficiency and low indel risk, which is needed for ex vivo editing of hematopoietic stem cells or T cells. Crop uses include prime editing to enhance crop traits (Fig. 7c), such as infectious disease and stress resistance, and yield. Specific gene corrections or introducing traits can be tailored with PrimeRoot and plant-optimised pegRNA, and they do not introduce transgenes, thus they allow applications that are compatible with regulatory frameworks in many regions. The transposition of effective regulatory components into crops or rewiring the gene networks can also be performed using large-fragment PE [3,71,72,105,106].

In animals, prime editing has emerged as a powerful tool for generating precise genetic modifications for functional genomics, disease modelling, and trait improvement (Fig. 7d). More recent results have also shown the effectiveness of prime editing in livestock and embryonic systems. For example, the introduction of defined mutations in sheep (FecB(B) and TBXT) and in porcine blastocysts (KCNJ5(G151R/+)) has been successfully achieved using prime editing with precision. Such works highlight the opportunities of prime editing to create agronomically beneficial traits and simulate disease-related mutations in large mammals, with great precision and minimal unintended genomic modification. It enables accurate introduction of point mutations, small insertions or deletions, and exon-level corrections in livestock and laboratory animals without inducing double-strand breaks. Prime editing has been successfully applied to create animal models of human genetic diseases, study gene function in vivo, and improve economically important traits such as growth, fertility, and disease resistance. Its high precision and reduced off-target effects make it particularly suitable for translational and agricultural animal research [[107], [108], [109], [110]]. PE can be applied in microbial biotechnology to optimise metabolic networks, establish biosynthetic pathways or to incorporate genetic logic units into bacteria or yeast. Compact CRISPR-based systems like mini-Cas12f editors broaden the genome editing possibilities for small spaces; dedicated prime editing systems are fruit being taken from bacteria for use in genome engineering. Here, computational design approaches can help to narrow down the list of possible pegRNAs and the number of constructs that need to be experimentally tested. However, empirical screening still is required as the result from editing the genome can be highly affected by genomic context and biological variability, and PE systems are used in new targets at a great speed. The extra flexibility of TwinPE plus PASTE allows for more precision in editing with the power of top precision aligned uses like programmable gene circuits, synthetic gene circuits, and developmental biology experiments. By virtue of its modularity, variants selected to be used in prime editing can be finely chosen depending on the specific needs of the application or genome (fidelity, size of the edit, delivery considerations, etc., and specific repair map features to preserve during delivery in the cell) [81,[111], [112], [113]].

## 2 Limitations, trade-offs, and comparative performance of prime editing systems

Prime editing technologies have different trade-offs in efficiency, precision, scalability, and compatibility. Preliminary systems, including PE2, are very accurate but cannot be edited efficiently because they lack strand bias. Conversely, PE3 enhances efficiency to a great extent through dual nicking, but at the cost of increased indel formation. The PE3b arrangement partly addresses the issue, as the second nick is temporarily controlled, and a compromise between efficiency and product purity comes into focus. MMR modulation schemes taught in PE4 and PE5 enhance editing efficiency and product purity in MMR-proficient cells. However, this raises concerns regarding genomic stability, especially in the therapeutic environment, due to transiently suppressed DNA repair pathways? Equally, variants with high-fidelity Cas tools tend to minimise off-target activity but can alter editing efficiency, which is a compromise between specificity and activity [2,40,72].

A large-fragment editing system will make additional design compromises. While accurate editing is possible with TwinPE no double-strand breaks are introduced, which requires complicated design and optimisation of pegRNA. High efficiency of integration of kilobases in PASTE also involves generating sites recognised by integrase, which may limit the plasticity of the genome. While successful in plant systems, PrimeRoot's use is limited by species-specific factors and challenges with delivery. AI-driven design of tools has streamlined pegRNA systems and eliminated the need for lab screening. However, they work best for the training set and may not be generalizable beyond these situations, particularly in poorly defined cell types or species. Combined with the other experiences, these findings indicate that none of the prime editing approaches is the optimal choice in all instances. Instead, selection of a system should be based on requirements for specific applications (e.g., edit size, accuracy, delivery conditions, cell repair environment) [[114], [115], [116]]. There are still more hurdles to overcome for the wider use of prime editing. Ideally, the efficiency of editing is high and has been reported in many of the engineered cell lines used, however, the efficiency will often be significantly decreased in primary cells, stem cells, embryos, and in vivo systems. This restriction becomes more noticeable for large fragment editing platforms like TwinPE, PASTE or PrimeRoot which need multiple editing components to act together. Moreover, editing results are still very much chromatin and DNA repair status- and location-dependent. This is because the efficiency of identical pegRNA designs can vary between different cell types as local chromatin structure, DNA methylation and activity of repair pathways can affect target accessibility and repair outcomes. Further points require attention are the use of PAM relaxed Cas variants like SpG and SpRY. These variants can greatly enlarge a PAM-expanded genome that can be targeted, but these larger and more flexible PAMs can also introduce more off-target sites, which may pose a challenge for guide design and further experimental testing. Finally, the immunogenicity of Cas proteins, reverse transcriptase domains, and delivery systems remains a potential challenge for therapeutic applications. Future studies will require improved delivery strategies, transient editor expression, and reduced-immunogenicity editor designs to facilitate clinical translation [42,58,117].

## 3 Future directions and conclusion

The future development of prime editing will focus on improving scalability, accuracy, and delivery capabilities. Recent studies have also explored strategies collectively referred to as regulated prime editing (rPE), which aim to improve editing outcomes by controlling cellular DNA repair processes during prime editing. In these methods, repair factors or intermediate stabilisation edits are modulated to stabilise the intended edit and reduce undesirable repair events. This may improve the efficiency and purity of multiple products from any future prime editing procedure by directing the repair of DNA breaks to the desired sequence. The other important move is expanding editing capability so that the genome can be rewritten at the kilobase scale without forming double-stranded intermediates. To achieve this, processive RT variants will be required, improved pegRNA scaffolds that can hold the template over long distances, and second-stage pegRNA paired-guide systems that can control the timing of the editing events [[118], [119], [120]]. These advancements will be in addition to curative exon substitution, regulatory unit addition, and total gene correction. Context-aware editing is another priority. A particularly important future direction is the development of chromatin-adaptive prime editing systems that dynamically respond to locus-specific chromatin states. Future editors may incorporate chromatin-sensing domains, epigenetic recruitment modules, or accessibility-guided design algorithms to modulate Cas binding, reverse transcription efficiency, and repair pathway engagement in a context-dependent manner. Such systems could reduce locus-to-locus variability and enable more consistent editing outcomes across heterochromatic and transcriptionally inactive regions. This approach extends prime editing from a sequence-targeting tool to a context-aware genome engineering platform. Optimising primers based on chromatin state, replication timing, or transcription timing maybe improve performance in difficult-to-access genomic regions. Editing variability could be reduced by targeting cells in the S-phase or by regulating cell-cycle-dependent repair mechanisms. The inducible, self-limiting editor design will be safer and enable accurate temporal control of editing activity in therapeutic applications. Delivery is also a major issue. Small prime editors, such as PEmini, split-PE systems, and Cas12f-based systems, will be improved to enable efficient in vivo delivery via AAV, LNP, or virus-like particles. The next steps for potential clinical applications are the use of tissue-specific promoters, microRNA-responsive pegRNAs, and immune-silent editor scaffolds [[121], [122], [123], [124]]. Another promising direction is the development of self-regulating prime editors capable of limiting their activity after successful editing. These systems may incorporate feedback-responsive pegRNAs, repair-coupled activation switches, or transient expression modules that deactivate the editor once the desired modification is achieved. Such self-limiting architectures could reduce prolonged exposure of genomic DNA to editing machinery, minimise off-target effects, and enhance safety for therapeutic applications. This represents a shift toward autonomous and controllable genome editing systems [2,125,126].

Computational approaches are expected to play an increasingly important role in prime-editing design through improvements in pegRNA prioritisation, editing-efficiency prediction, and editing-outcome modelling. Future advances will likely benefit from larger and more diverse experimental datasets, enabling improved model performance across different genomic loci, cell types, and organisms. Integration of chromatin accessibility, DNA repair pathway activity, and sequence-context information may further enhance predictive accuracy. However, automated optimisation frameworks, reinforcement-learning approaches, and closed-loop experimental systems remain largely conceptual and have not yet been widely validated for prime-editing applications. Therefore, current computational tools should be regarded as decision-support systems that complement, rather than replace, experimental optimisation and validation, significantly accelerating the design build test cycle [88,[127], [128], [129]].

A central theme emerging from recent advances is the convergence of molecular engineering, RNA design, and computational prediction into an integrated editing ecosystem. Rather than incremental improvements in individual components, the field is transitioning toward system-level optimisation in which enzyme activity, RNA architecture, and DNA repair pathways are co-designed. This shift marks a transition from descriptive tool development to predictive genome engineering. In conclusion, prime editing has evolved into a highly versatile genome engineering tool through advances in Cas proteins, reverse transcriptase, pegRNA design, and nicking strategies. From early systems like PE1-PE3 to improved versions such as PEmax and large-fragment approaches like TwinPE and PASTE, both efficiency and editing scope have expanded significantly. Innovations, including flexible PAM recognition, compact Cas variants, and AI-guided design, have further enhanced its applicability. Together, these developments position prime editing as a powerful and programmable platform for precise genome modification across medicine, agriculture, and synthetic biology [116,130,131].

## Informed consent statement

Not applicable.

## Author contributions

Waqar Muhammad contributed to the conceptualisation, investigation, and drafting of the original manuscript. Xi Cao, Aziz Umar and Khan Nauman provided critical input through review, editing, data curation, and validation. Ziao Jiao and Junjie Liu contributed to the figure drawings. Xiaolong Wang reviewed the draft and provided many constructive suggestions. Kun Xu as the corresponding authors, supervised the project, secured funding, monitored its progress, and were responsible for the final review and editing of the manuscript. All authors have read and approved the final version of the manuscript.

## Funding

This work is supported by the Agriculture Science and Technology Major Project and the Biological Breeding-Major Projects (grant nos. 2023ZD04074 and 2023ZD04051).

## Declaration of competing interest

The authors declare that they have no known competing financial interests or personal relationships that could have appeared to influence the work reported in this paper.
